# Hemostatic nanoparticles increase survival, mitigate neuropathology and alleviate anxiety in a rodent blast trauma model

**DOI:** 10.1038/s41598-018-28848-2

**Published:** 2018-07-13

**Authors:** W. Brad Hubbard, Margaret Lashof-Sullivan, Shaylen Greenberg, Carly Norris, Joseph Eck, Erin Lavik, Pamela VandeVord

**Affiliations:** 1 Biomedical Engineering and Mechanics, Virginia Tech, Blacksburg, VA USA; 2 Department of Biomedical Engineering, Case Western Reserve University, Cleveland, OH USA; 3 Translational Biology, Medicine, and Health, Virginia Tech, Blacksburg, VA USA; 4 Department of Chemical, Biochemical and Environmental Engineering, University of Maryland- Baltimore County, Baltimore, MD USA; 5 Research Services, Salem VAMC, Salem, VA USA; 6 Present Address: Spinal Cord and Brain Injury Research Center (SCoBIRC), University of Kentucky College of Medicine, Lexington, KY USA

## Abstract

Explosions account for 79% of combat related injuries and often lead to polytrauma, a majority of which include blast-induced traumatic brain injuries (bTBI). These injuries lead to internal bleeding in multiple organs and, in the case of bTBI, long term neurological deficits. Currently, there are no treatments for internal bleeding beyond fluid resuscitation and surgery. There is also a dearth of treatments for TBI. We have developed a novel approach using hemostatic nanoparticles that encapsulate an anti-inflammatory, dexamethasone, to stop the bleeding and reduce inflammation after injury. We hypothesize that this will improve not only survival but long term functional outcomes after blast polytrauma. Poly(lactic-co-glycolic acid) hemostatic nanoparticles encapsulating dexamethasone (hDNPs) were fabricated and tested following injury along with appropriate controls. Rats were exposed to a single blast wave using an Advanced Blast Simulator, inducing primary blast lung and bTBI. Survival was elevated in the hDNPs group compared to controls. Elevated anxiety parameters were found in the controls, compared to hDNPs. Histological analysis indicated that apoptosis and blood-brain barrier disruption in the amygdala were significantly increased in the controls compared to the hDNPs and sham groups. Immediate intervention is crucial to mitigate injury mechanisms that contribute to emotional deficits.

## Introduction

Traumatic injury is the leading cause of death for both men and women between the ages of 5 and 44 worldwide^1^, and blood loss is the primary cause of death at acute time points post injury^2,3^. Immediate intervention is one of the most effective means of minimizing mortality associated with severe trauma^4^, and yet the only available treatments in the field are pressure dressings and absorbent materials which are effective for exposed wounds, but cannot address internal injuries. 79% of casualties on the battlefield result from explosions; these explosions create pressure waves that cause complex blast injuries, which can involve multiple injuries including traumatic brain injury (TBI) and internal bleeding^3^.

Coagulopathy is presented in approximately 65% of cases after terrorist bombings with mortality reaching 50%^5^. In a study conducted in the U.K., less than 50% of Soldiers diagnosed with primary blast lung injury (PBLI), the most common fatal blast injury, survived to reach a medical facility^6^. Mortality from PBLI sustained in recent combat has also been reported in US troops^7^. Following whole body blast exposure, the primary concern is to increase acute survival. Secondarily, chronic outcomes, such as mental health impairment, can be mitigated early if treatment addresses acute neuropathology^8^. Anxiety-like symptoms associated with exposure to blast have been reported clinically in Soldiers after deployment and are comorbid with post-traumatic syndrome disorder (PTSD)^9,10^. Reports have estimated that an average of 25% of patients (up to 60%) with TBI had generalized anxiety disorder, five times that of the normal population^11–15^. The amygdala, a region of the brain that synthesizes cognitive and emotional inputs, is crucial in processing fear and negative emotions. Anxiety has been reported to coincide with overt damage in the amygdala in Vietnam Veterans, suggesting that changes in the amygdala are critical to the elevated levels of anxiety seen after blast trauma^16^. Collectively, blast-induced traumatic brain injury (bTBI) in military personnel leads to long-term effects on the nervous system and is widely recognized as a risk factor in developing neurodegenerative disease and mental health concerns^17^.

To address the therapeutic need for blast trauma, we designed hemostatic nanoparticles (hNPs) to stop bleeding and reduce inflammation. hNPs lead to an increase in survival in a mouse model of blast injury^18^, but survival is only part of the challenge. With the advent of improvised explosive devices, there has been a significant rise in associated brain injuries. We wanted to engineer a variant of the hemostatic nanoparticles to address this significant challenge. We hypothesized that nanoparticles that could reduce bleeding as well as deliver an anti-inflammatory to the injured tissues might improve systemic outcomes. To this end, we devised hNPs that deliver dexamethasone^19^. Dexamethasone is a potent steroid that can dramatically reduce inflammation in the brain after injury^20–22^.

We expect that intravenous administration of hemostatic nanoparticles delivering dexamethasone (hDNPs) would reduce bleeding and inflammation and improve survival after blast trauma. We asked whether the potential reduction in hemorrhage and inflammation would lead to better neurological outcomes. Recent work looking at neurological damage following blast demonstrated microhemorrhaging, edema and blood-brain barrier (BBB) disruption^8,23–26^. We hypothesize that hDNPs could mitigate these negative outcomes. To test this, we exposed rats to full body blast trauma and administered hDNPs or control treatments and compared behavioral pathology. We characterized the animal’s survival, recovery via oxygen saturation, and anxiety-like behavior using an open field tracking system. We correlated the behavioral findings with histological measures of neural degeneration, BBB integrity, inflammatory cell numbers, and gliosis within the amygdala. Administration of hDNPs resulted in reduced anxiety-like behavior and greater neural preservation following blast injury and promote further research to advance this viable treatment option.

## Results

### Hemostatic nanoparticles improved survival

**Figure 1:**
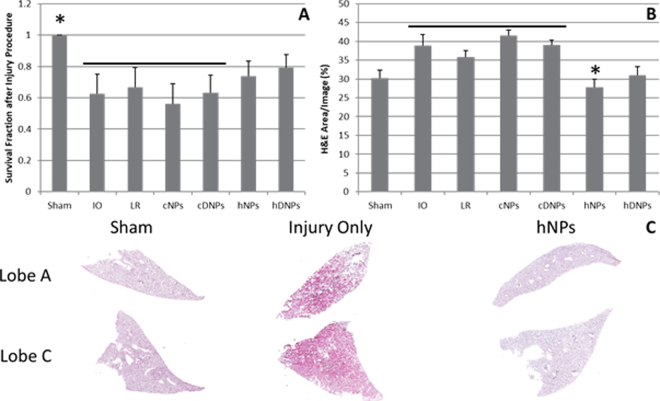
(**A**) Survival fraction after blast exposure. The sham group was significantly different from IO, LR, cNPs, and cDNPs groups (*p < 0.05). (**B**) H&E percent area from internal hemorrhaging in lung cross-sectional images. The hNPs group was significantly different from IO, LR, cNPs, and cDNPs groups (*p < 0.05). (**C**) Representative cross-sectional lung lobe images from Sham, IO, and hNPs groups.

PBLI is a highly lethal injury associated with mortality levels ranging from of 11% to 55% according to some reports, with this variance likely due to differences in severity^27,28^. Hemostatic nanoparticles led to improved survival outcomes compared to controls. Survival percentage was significantly lower (p < 0.05) in the control groups: IO (Injury Only), LR (Lactated Ringer’s), control nanoparticles (cNPs), and control dexamethasone-loaded nanoparticles (cDNPs) groups compared to the shams. Control groups, cNPs and cDNPs, were injected nanoparticles conjugated with the peptide GRADSP, which lack binding to the active hemorrhaging site. However, the hNPs and hDNPs groups were comparable to the shams (Fig. 1A) suggesting that the hemostatic nanoparticles (hNPs and hDNPs) led to increased survival following blast trauma.

### Internal hemorrhaging in the lungs was mitigated by hNPs

H&E staining of the lungs was conducted to investigate the impact of hNPs on internal hemorrhaging. Images in Fig. 1C show fewer red blood cells in lung after hNPs treatment compared to IO, which displays widespread internal hemorrhaging, consistent with blast injury. The hNPs group had significantly fewer red blood cells (p < 0.05) compared to IO, LR, cNPs, and cDNPs groups (Fig. 1B) indicating that less hemorrhaging occurred with the treated animals.

### hDNPs reduced anxiety after blast injury

**Figure 2:**
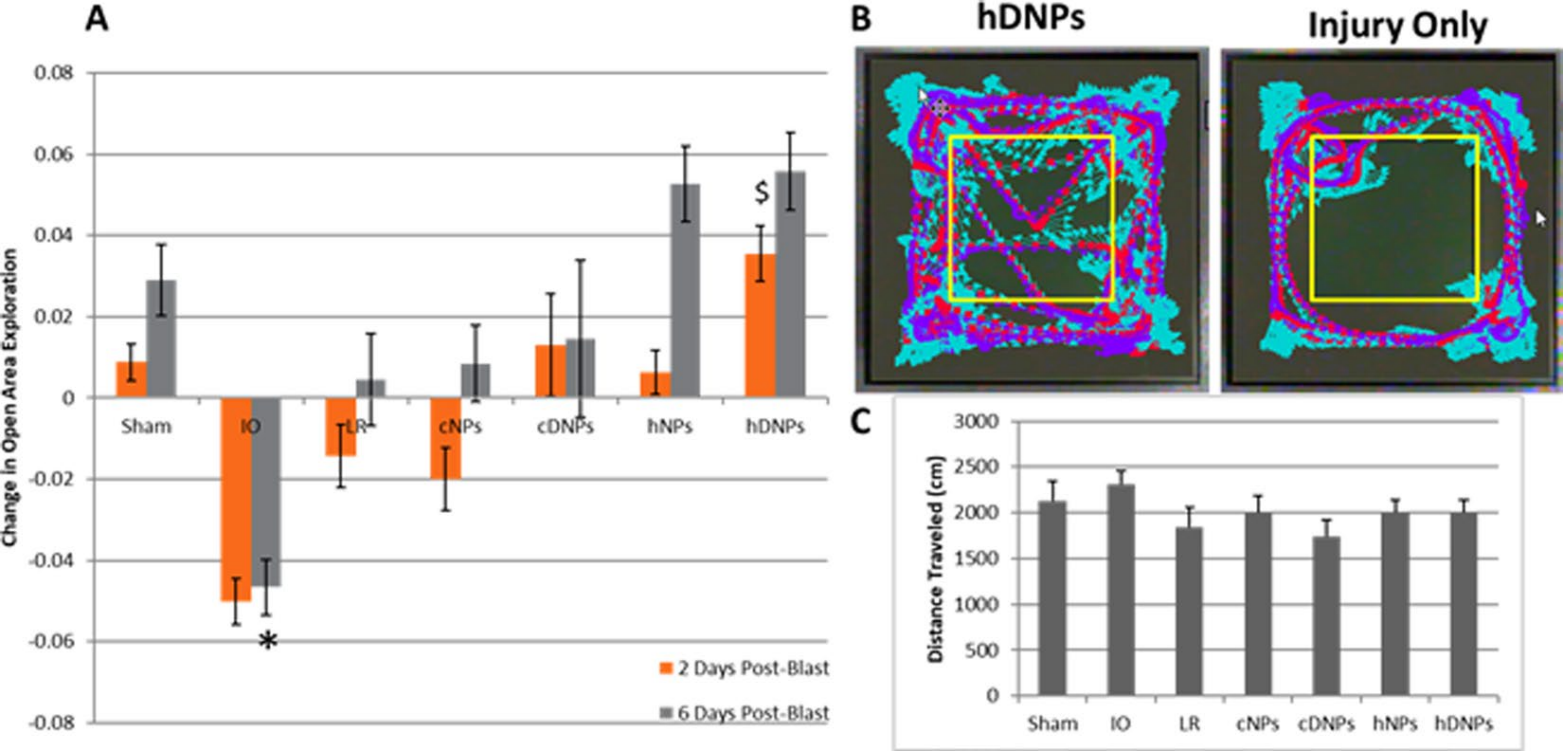
(**A**) Rodents demonstrated changes in open area exploration over time after blast. At two days post-blast, hDNPs group was significantly different from IO, LR, and cNPs groups ($p < 0.05). At six days post-blast, the IO group was significantly different from sham, hNPs, and hDNPs groups (*p < 0.05). (**B**) Representative rodent tracking for five minutes at six days post-blast in the hDNPs and IO groups. (**C**) Distance traveled during the open field exploration test. No significance was determined between the groups suggesting normal motor activity following blast.

Two days after blast exposure, rodents were placed in the open field arena and allowed to explore for five minutes. Rodents are instinctively curious but also can exhibit tracking behavior along the walls, known as thigmotaxic behavior. Animals with anxiety-like behavior exhibit more time at walls compared to sham animals^29,30^. The hDNPs group exhibited significantly increased time exploring the center of the arena (p < 0.05) compared to cNPs, LR, and IO groups (Fig. 2A) at two days post-blast without any differences in distance traveled or average velocity over the five-minute behavioral test (Fig. 2C). At six days following blast exposure, the sham, hNPs, and hDNPs groups displayed significantly higher exploration (p < 0.05) of the open center of the arena compared to IO (Fig. 2A). Representative tracking for a hDNPs-treated rodent (Fig. 2B; left) versus an IO rodent (Fig. 2B; right) elucidates this difference.

### hDNPs reduced apoptosis after blast injury

**Figure 3:**
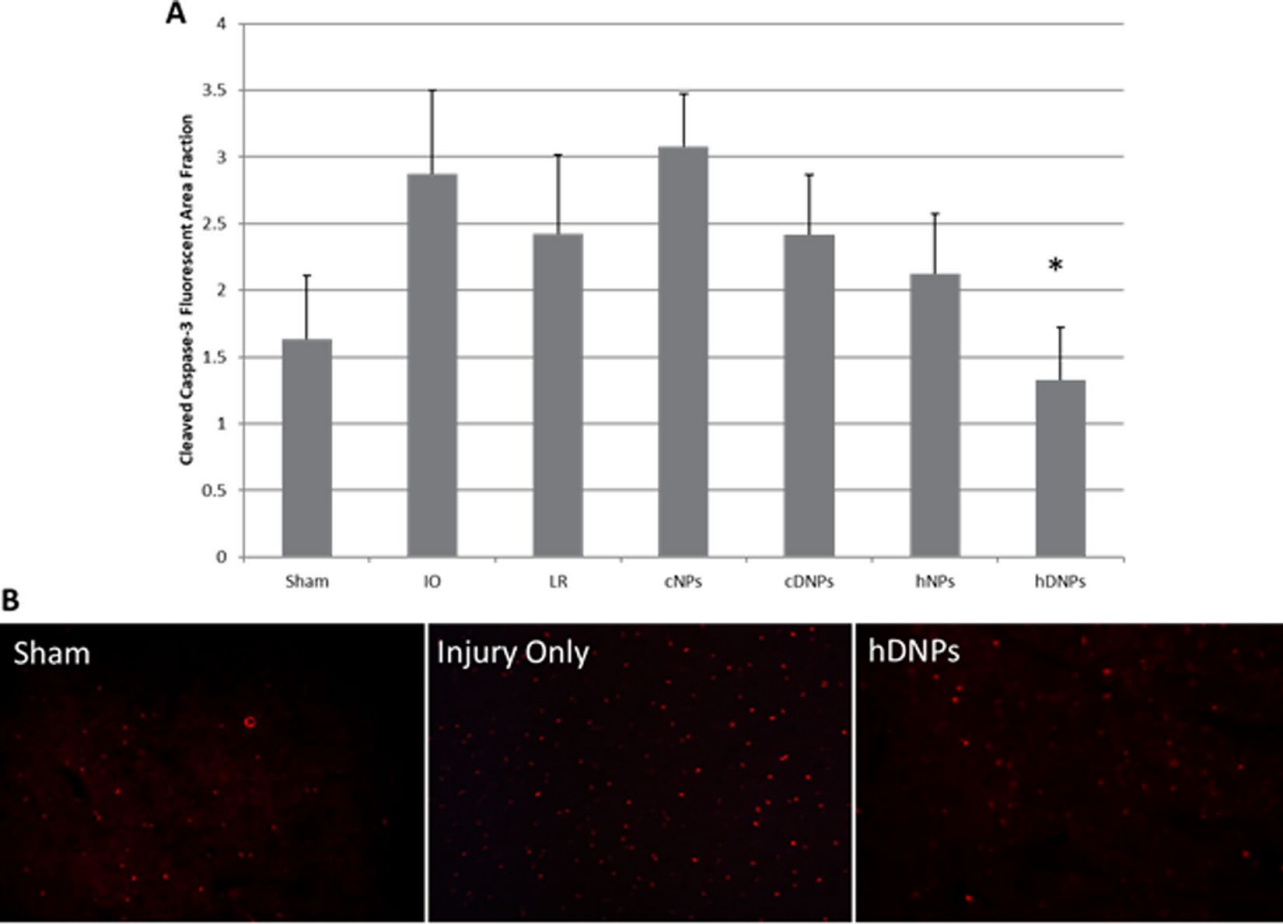
(**A**) Fluorescent area for cleaved caspase-3, an apoptotic marker. At seven days post-blast, the hDNPs group was significantly different from IO and cNPs groups (*p < 0.05). (**B**) Representative images from the amygdala for cleaved caspase-3 from hDNPs, IO and sham animals.

Cleaved caspase-3, a marker for early stage apoptosis, was significantly diminished (p < 0.05) in the amygdala with hDNPs treatment as compared to the cNPs and IO groups (Fig. 3A). Representative images of cleaved caspase-3 levels in the amygdala show more apoptosis occurring in IO (Fig. 3B) compared to hDNPs suggesting the administration of hDNPs may limit apoptosis.

### hDNPs restore microglia levels after blast injury

**Figure 4:**
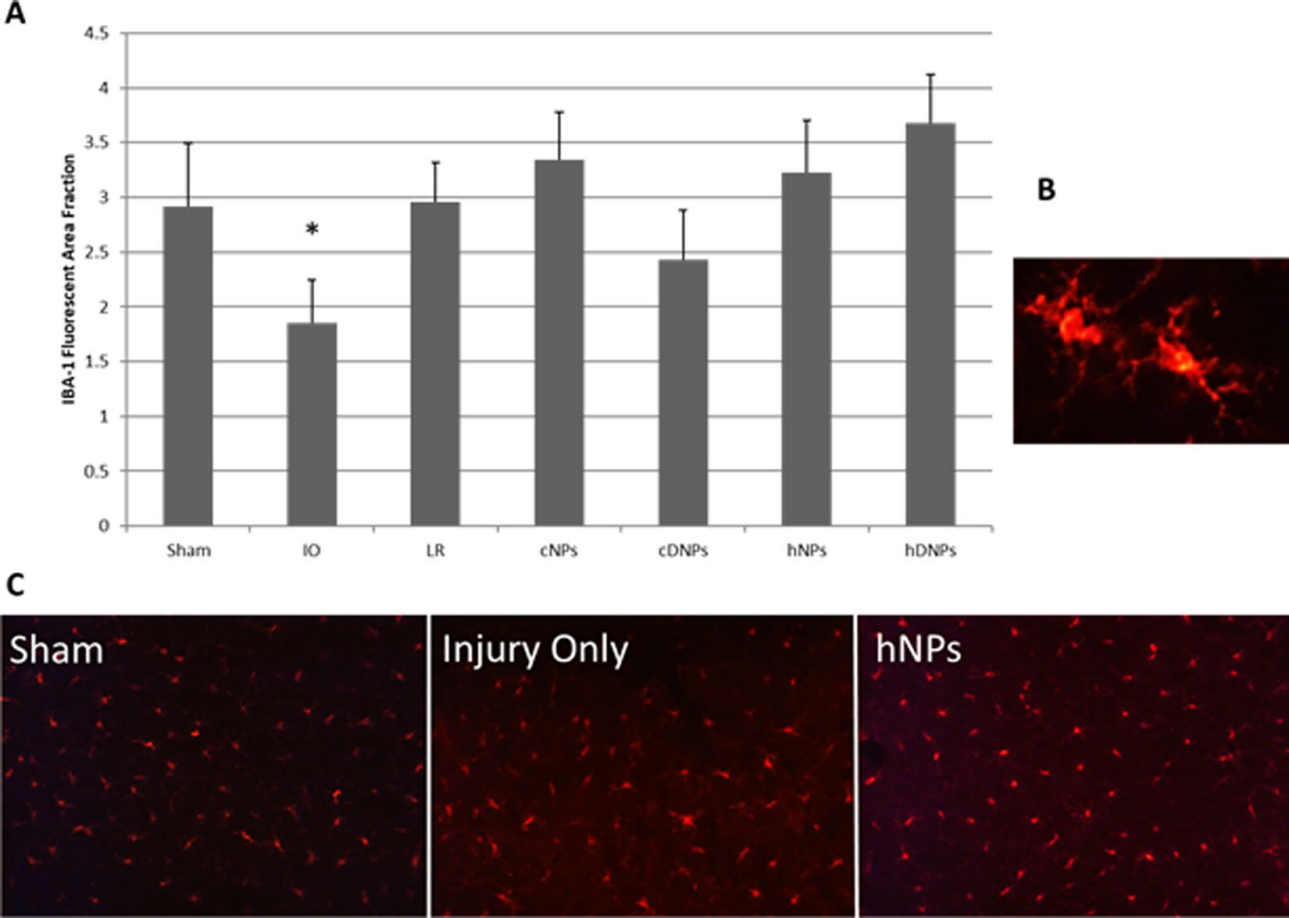
(**A**) Fluorescent area for IBA-1. At seven days post-blast, the hDNPs group was significantly different from IO and cNPs groups (*p < 0.05). (**B**) Example of process retraction in the Injury Only group. (**C**) Representative images of the amygdala for IBA-1 from hNPs, IO, and sham animals.

IBA-1, marking microglia within the amygdala, was significantly decreased with IO as compared to the hDNPs (p < 0.05) (Fig. 4A). Lower fluorescent area is likely indicative of process retraction (Fig. 4B) in the Injury Only group. Representative images of IBA-1 in the amygdala further depicting process retraction occurring in the IO group (Fig. 4C) compared to hDNPs, showing a possible morphology shift due to blast that is avoided with hDNPs treatment.

### Hemostatic nanoparticles reduced astrogliosis after blast injury

**Figure 5:**
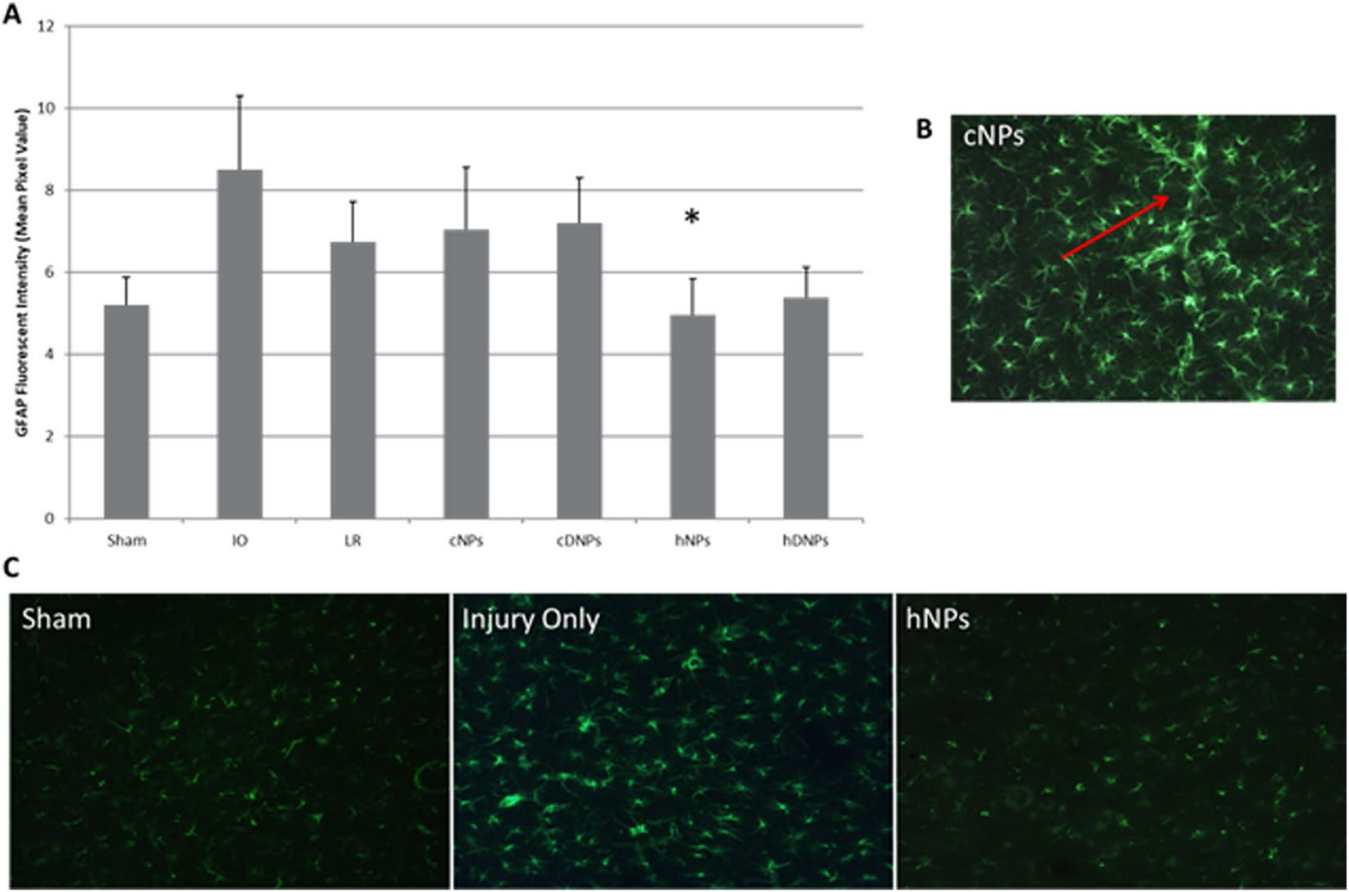
(**A**) Fluorescent intensity of GFAP staining, a marker indicating astrocytic activation. At seven days post-blast, hNPs was significantly different from the IO group (*p < 0.05). (**B**) Astrocytic reactivity was observed surrounding vasculature in the cNPs group. (**C**) Representative images from the amygdala for GFAP from hNPs, IO and sham animals.

Astrogliosis within the amygdala was significantly decreased (p < 0.05) after hNPs treatment as compared to the IO (Fig. 5A). The other control groups also presented with elevated GFAP levels, though not significantly different from hNPs or hDNPs. Control groups are displayed reactive astrogliosis surrounding brain microvasculature (Fig. 5B). Images of GFAP staining in the amygdala depicted higher astrocytic activation with the IO compared to hNPs (Fig. 5C), demonstrating that treatment with hNPs reduces GFAP expression.

### Blood-brain barrier integrity was improved in hDNPs groups after blast injury

**Figure 6:**
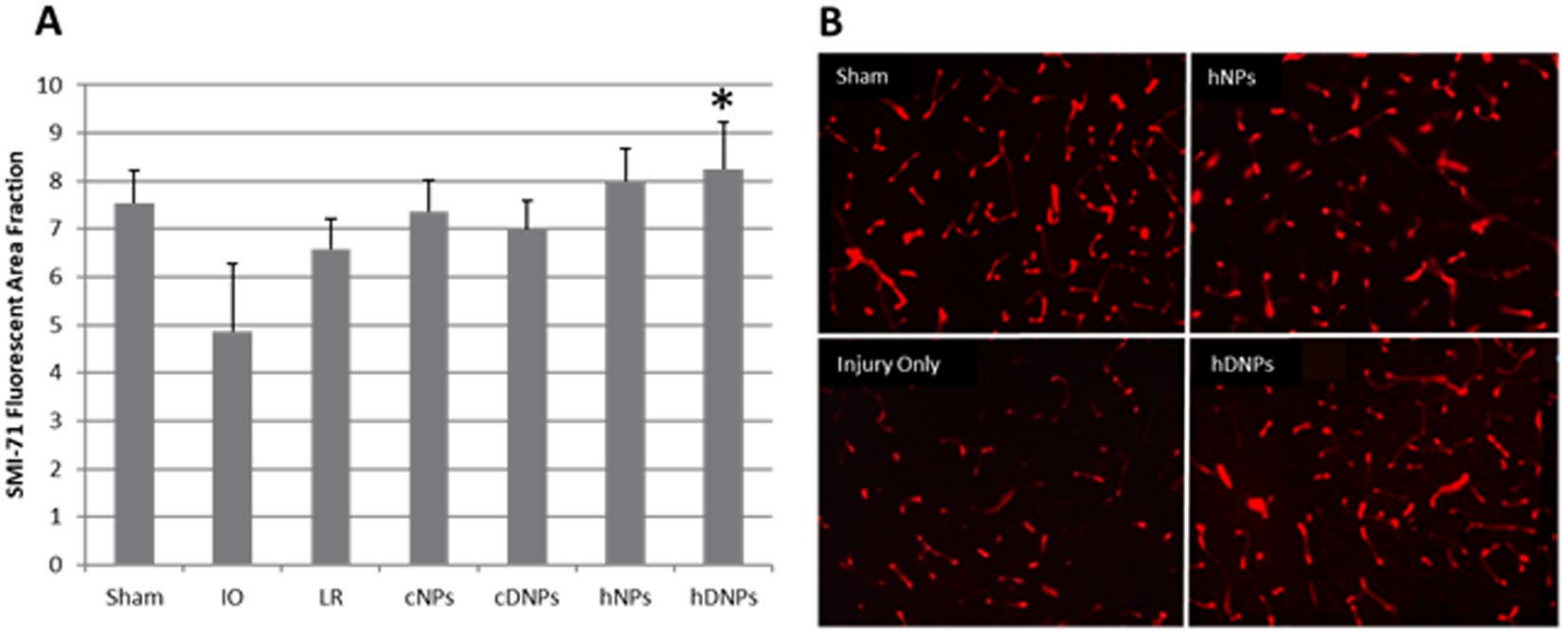
(**A**) Fluorescent area of SMI-71, a marker for BBB integrity. At seven days post-blast, hDNPs group was significantly different from the IO group (*p < 0.05). (**B**) Representative images of the amygdala from hDNPs, hNPs, IO, and sham animals. IO sections contained lower number of SMI-positive vessels compared to hDNPs and sham groups.

SMI-71 is an established antibody against rat endothelial barrier antigen (EBA) that marks the functionality of the BBB^31^. This antibody binds to EBA, which is not present in vessels with BBB disruption. The delivery of hDNPs following blast injury led to greater expression of the marker for BBB integrity at seven days post blast compared to the IO group (p < 0.05) (Fig. 6A). Immunostaining for EBA showed a greater density of EBA vessels in the sham and hDNPs groups compared to the IO group (Fig. 6B). This suggests that administration of the hDNPs protects or promotes more rapid reformation of the BBB after blast trauma.

### Dexamethasone loading was crucial to alleviating vascular endothelial growth factor expression

**Figure 7:**
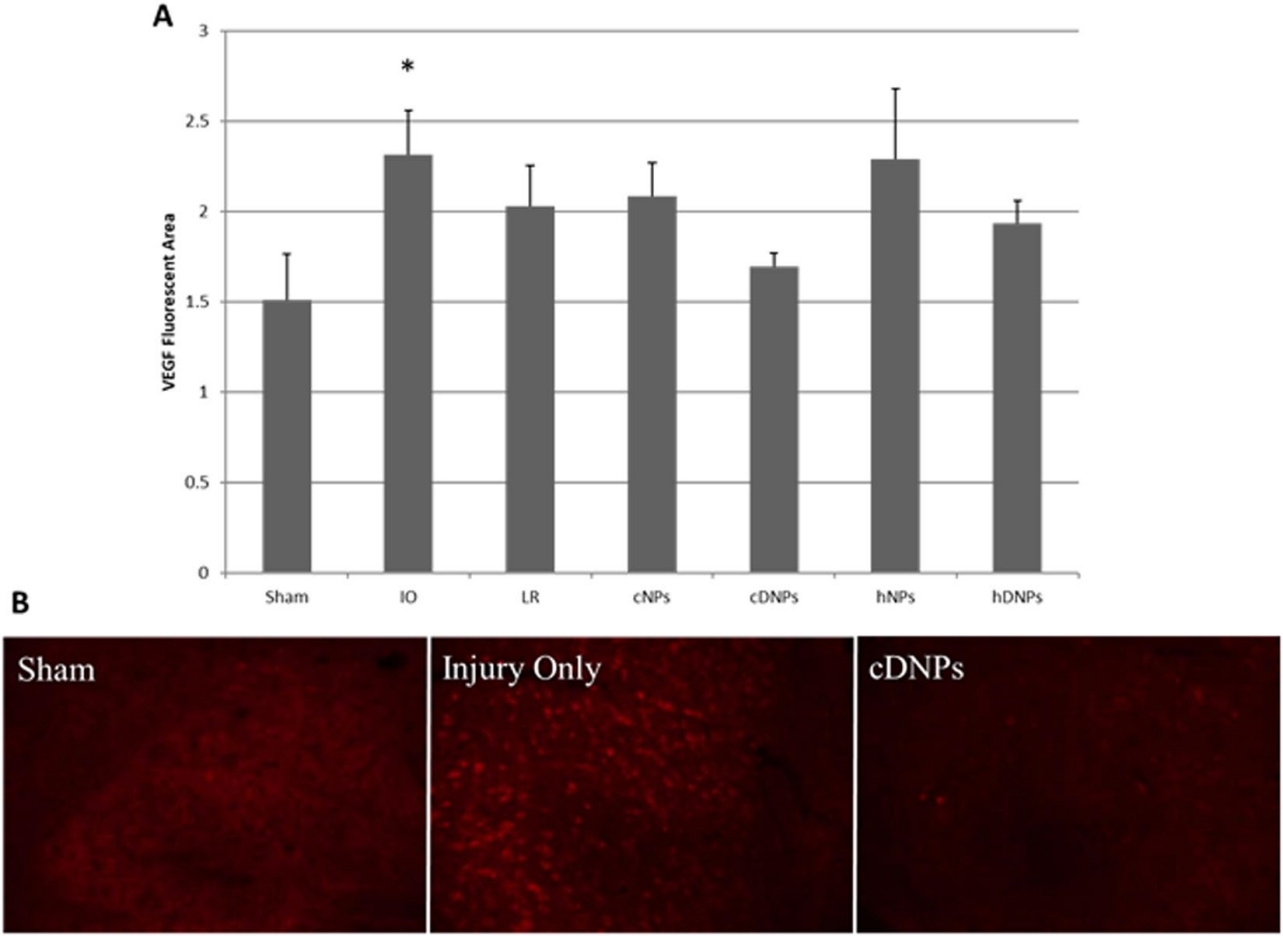
(**A**) Fluorescent intensity of VEGF staining. At seven days post-blast, Injury Only was significantly different from the sham and cDNPs group (*p < 0.05). (**B**) Representative images of VEGF in the amygdala from cDNPs, IO and sham animals.

Vascular endothelial growth factor (VEGF) expression within the amygdala was significantly increased (p < 0.05) with IO treatment as compared to the sham and cDNPs groups (Fig. 7A). Representative images of VEGF in the amygdala depict cells expressing high levels of VEGF in the IO group (Fig. 7B) compared to cDNPs.

## Discussion

The nanoparticles used in this study were functionalized with the glycine-arginine-glycine-aspartic acid-serine (GRGDS) peptide to target activated platelets. When platelets are activated, they expose the glycoprotein IIb/IIIa receptor^32,33^. While the RGD peptide is extremely common, the simplicity of the motif coupled with its affinity to activated platelets makes it an attractive choice for modulating hemostasis. While these particles have been shown to mitigate internal bleeding^18,34,35^, this is the first study examining secondary neuroprotective effects. Full nanoparticle properties and characterization were adapted from Hubbard *et al*.^19^. In brief, scanning electron microscope (SEM) imaging was utilized to demonstrate that the spherical nanoparticle was approximately 500 nm in size, which was validated by dynamic light scattering (DLS). Dexamethasone loading was confirmed via NMR (δ = 1.68) and the produced particles had a final loading of 22 ± 1 micrograms of dexamethasone per milligram of particles. The release profile of dexamethasone *in vitro* shows an initial burst followed by slow release over months after injection, though the release *in vivo* may be slower^19^.

Survival was significantly improved following blast trauma with the administration of hemostatic nanoparticles whether or not they encapsulated dexamethasone (Fig. 1A). This expands upon the work seen previously using hNPs following blast trauma in mice^18^. Acute mortality after blast exposure likely stems from severe lung damage as the pressure wave associated with blast preferentially impacts air-filled organs^36,37^. Hemorrhage in the lungs, signified by red blood cell (RBC) infiltration, was reduced with hNPs (Fig. 1B). Our previous study examining hDNPs after blast injury found similar results, but this data supports therapeutic ability of hNPs, as well, in this rodent model of blast polytrauma^19^. For internal hemorrhage reduction, the therapeutic benefit was dependent on the hemostatic portion of the nanoparticle rather than dexamethasone release, as expected. Both survival and hemorrhage data support that the hemostatic functionality of the nanoparticles is crucial to improving acute outcomes.

There have been several clinical studies reporting that Veterans suffering from major depressive disorder (MDD) following blast exposure, as well as individuals with PTSD, displayed amygdalar hyperactivity^9,38^. Prevalence of PTSD symptoms, self-reported and scored by clinicians, was higher in Veterans with a history of TBI^39^. bTBI has been associated with anxiety disorders and symptom crossover with combat-associated PTSD^40,41^. Neurological symptoms, especially those with emotional impairment, are a major concern for quality of life in returning Soldiers and Veterans. Directing therapeutics towards anxiety mitigation is needed for military personnel and civilians after a traumatic event.

Assessments of anxiety-like behavior have been used experimentally in animal models of blast-induced neurotrauma. Standard tests such as the elevated plus maze, light/dark box, and open field assessment all suggest that anxiety-like behavior is common following blast exposure^42,43^. The avoidance of the center in an open arena strongly suggests anxiety-like behavior following blast trauma, and the administration of the hDNPs appears to reduce this anxiety-like behavior (Fig. 2). hDNPs-treated rodents showed fewer signs of anxiety-like behavior following blast in the open field assessment. Anxiety outcomes are not unique to blast polytrauma as it is observed after isolated bTBI^42,44^. The presence of anxiety also seems to be independent of the level of blast overpressure exposure. Anxiety was seen in open field activity at seven days following 25–40 psi blast exposure as well as after low-level blast exposure^43,45^. A study examining the relation of blast exposure to PTSD in a rat model found increased anxiety parameters from repeated mild exposure, in the absence of psychological stressors^46^. Chronic levels of increased stathmin-1 were reported at eight months after repeated mild blast exposure, which is responsible for fear responses^46^. The open field test is a standard test to measure anxiety-like behavior^30^. We observed that animal movement and distance traveled were not significantly different between any groups (Figure 2C), while there was a therapeutic effect on exploration in the center of the open arena. The reduction in elevated thigmotaxia, representative of anxiety, at multiple time points (2 and 6 days) after injury in the animals treated with hDNPs is a promising result for the therapeutic to have an impact on neurological recovery.

This is the first study to provide evidence that subacute outcomes of blast injury also improve following hDNPs or hNPs administration. To corroborate our findings of anxiety, we have focused our pathological investigation to the amygdala. This model of blast polytrauma has previously shown exacerbated neuropathology in the amygdala^47^ and has been linked to the presentation of anxiety-like behavior in both clinical and pre-clinical studies^9,38,42,44,46^. Cell death is often the result of injury cascades and has been documented globally within the brain after blast injury^42,44,48^. In a murine model of blast neurotrauma, neuronal reduction and glial activation were noted within the amygdala^45,49^. However, with the benefit of injury site targeting and hemostasis, these injury mechanisms are mitigated with the hNPs and hDNPs treatments. Although this model was not found to have significant levels of neurodegeneration measured by Fluoro-Jade-C positive cells in the amygdala, cell death was observed. We hypothesis that neurons are undergoing apoptosis (Fig. 3), which may have an effect on the observed neurological deficits.

Microglia alteration after blast injury has been shown to occur and have a distinct role in bTBI pathology^50^. Specific cell phenotypes can guide the recovery process after injury, eliciting necessary neuro-recovery^51^. Given this dual role (beneficial/detrimental) that microglia display after injury, it is possible that microglial presence at this time point after injury could provide a necessary response after blast trauma, which is lacking after IO (Fig. 4)^52–55^. Vascular dysfunction after blast has been reported with activated microglia morphologies, including ameboid cells with retracted processes^56^. With this, it is also possible that this activated morphology with retracted processes and lower IBA-1 expression occurs in the amygdala in this blast polytrauma model, explaining lower fluorescent intensity after blast only.

Secondary mechanisms, such as neuroinflammation and BBB dysfunction, have been shown to contribute to subacute and chronic neurological dysfunction as activation of astrocytes and microglia has been reported in models of bTBI^42,48,57^. Perez Polo *et al*.^58^ found increased microglia activation in the amygdala at six hours and 30 days after injury. Similar to our results (Figs 3–5), elevated levels of activated glia and apoptosis have also been reported at seven days post-blast in the amygdala^42^, demonstrating on-going injury cascades in the subacute stages. During blast injury, the BBB is disrupted and this decreased integrity permits the influx of molecules and cells that can further exacerbate injury leading to secondary degeneration^59^. Studies have reported that agents enhancing recovery of the BBB are associated with greater neuroprotection and better outcomes following TBI^60,61^.

BBB disruption can be caused by and lead to a myriad of molecular cascades^61^. This cyclical relationship is elucidated with the finding that BBB disruption is biphasic, occurring at multiple time points after traumatic brain injury^24,62^. BBB disruption has been reported in many blast studies, suggesting that it can be due to primary or secondary injury mechanisms^23,25,26^. Mechanisms of secondary BBB leakage with impairment of tight junctions and pericytes have been connected with activation of aquaporin-4 and matrix metalloproteinases (MMPs)^25^. The size of BBB leakage after blast has been investigated by several studies, specifically one model of blast polytrauma, identifying immunoglobulin (IgG) infiltration (160 kDa) post-blast^24,63^. BBB breakdown has been shown to be a major factor in development of neurologic disease due to altered neurovascular dysfunction^64^. Activated glia and neuroinflammation is hypothesized to be a result of BBB disruption^65^. Transport of larger molecules, not allowed under normal conditions, across the barrier can be costly for a supporting environment of neural recovery after injury. Combatting capillary rupture within the cerebrovasculature after high intensity blast exposure at an early time point is crucial for limiting the amount of neurologic consequences developed at a later stage^8^.

It has been shown that there can be BBB disruption and increased permeability due to hypoxia^66^, which occurred acutely in this full-body polytrauma model^19^. Astrocytes play a large role in BBB health, as end-feet coverage is crucial for proper function and regulation with compromise leading to various disorders^67^. While BBB disruption has been determined to be biphasic in TBI, the time course in this polytrauma model is uncertain, except for disruption at seven days post-blast^62^. SMI-71, as a marker for BBB disruption, has been correlated with FITC-albumin infiltration^31^. Lower number of EBA+ vessels and stained vessel area are associated with regions of BBB dysfunction. Reduction of BBB disruption in the animals treated with hDNPs suggests a mitigation of on-going pathology and quick recovery of BBB function at seven days. This highlights the ability of hDNPs to address initial primary injury to the BBB and persistent secondary opening of the BBB through dexamethasone release.

Previously in this model, hypoxia-inducible factor-1α (HIF-1α) and VEGF markers have shown to be upregulated after blast polytrauma^47^. Additionally, levels of HIF-1α and VEGF in plasma were elevated at 42 days post-blast in a model of repeated mild TBI demonstrating long lasting effects of hypoxia and chronic regulation of vascular permeability by VEGF^68^. In a blast TBI model, HIF-1α was elevated in serum at three days and one week post-blast, as well as after low intensity blast exposure in the serum at two hours, one day, one week, and one month post-blast, showing constant upregulation^68,69^. Five days after initiation of injury, astrocyte reactivity was increased in the amygdala in a rat model of repeated mild TBI along with increased levels of VEGF; both factors can be upregulated by hypoxic conditions and can regulate vascular permeability^44^. Hypoxia is the principal regulator of VEGF expression^70,71^. VEGF, as a downstream marker, has caused BBB dysfunction in models of TBI^72,73^. Lower levels of HIF-1α as compared to controls were seen after blast polytrauma and hDNPs treatment in this study although not significant (unpublished data). It is possible that HIF-1α levels peak before seven days, as downstream VEGF was elevated at this time point. VEGF, a factor in BBB disruption, levels were lower in treatment groups with dexamethasone, highlighting one potential avenue of BBB restoration (Fig. 7).

The therapeutic effect of dexamethasone has been previously investigated in other injury models^19,22,74^. A review by Obermeier *et al*. deemed glucocorticosteroids (GC) as the only applicable BBB therapeutic^59^. Dexamethasone, an anti-inflammatory GC, inhibits MMP and consequently improves vessel wall integrity by preserving BBB components^75^. In an *in vitro* model of primary blast injury, BBB restoration occurred *via* GC receptor signaling by dexamethasone^60^. Dexamethasone also attenuated astrocytosis around neural prosthetic devices after peripheral injection and local delivery through polymers^76^. Hemostatic nanoparticles have been used in treatment of acute hemorrhaging in injury models^18,34,35^. Since animals treated with cDNPs had higher levels of astrogliosis and BBB disruption compared to the hDNPs-treated animals, the targeting ability of the nanoparticles is a key event for successful management of the complex injury. In addition to providing enhanced coagulation, hemostatic nanoparticles also release dexamethasone at the injury site, pinpointing areas that need anti-inflammatory therapy that can repair BBB damage.

In the current study, hDNPs provided benefits over hNPs in terms of reducing BBB dysfunction and apoptosis. Dexamethasone has promising therapeutic benefit in restoring BBB after trauma and likely played a role in BBB recovery in this model^22,60^. One mechanism of indirect therapeutic benefit is the restoration of oxygen saturation by administration of hNPs acutely after blast exposure^19^. This could potentially reduce secondary mechanisms, such as upregulation of HIF-1α, in the brain after injury^47^. Although there is no significance in HIF-1α levels at seven days post-blast, we hypothesize that these levels differ in hNPs compared to controls in the acute injury state.

While hNPs have a role in reduction of lung injury, there is also potential direct therapeutic mechanism in the brain. The model in this study consists of a higher blast exposure in a lateral orientation, which is understudied in terms of the effect on specific brain regions. Overpressure dependence also is known to play a role in neurologic impairment, elevated glial activity and neurodegeneration following blast^42,48,77^. In a model of primary blast-induced neurotrauma, lateral blast exposure, similar to the model examined in this study, to the rodent head resulted in lesions in both hemispheres of the brain^23^. This finding was dependent on peak blast overpressure but independent of time point, demonstrating on-going BBB disruption in the amygdala^23^. Primary interaction of hemostatic nanoparticles in the amygdala is proposed to reduce microhemorrhaging, or capillary rupture of BBB, in order to promote BBB recovery at seven days and mitigate neuroinflammation measured by astrogliosis.

Since our assessment of the therapeutic effect was only evaluated at one week after injury, further studies are vital for determining the dynamic recovery phase with hDNPs administration. To aid in full understanding of hDNPs, future studies will examine the *in vivo* dexamethasone release profile as well as the pharmacokinetics of dexamethasone in conjunction with the injected nanoparticles *in vivo*. While examining secondary neuropathology proved to be an important aspect of this study, looking at acute biomarkers to ascertain specific mechanisms of therapeutic benefit after polytrauma is needed. In order to garner the full therapeutic benefit, more time points will be assessed after injury and therapeutic intervention. Hypoxia has been shown to extend until at least three hours post-blast after PBLI^78,79^. Examining the first emergence of HIF-1α after injury (within hours post-blast) and the daily progression will demonstrate if this pathway is crucial to injury progression and a therapeutic target. BBB disruption can be a primary and/or secondary pathology after blast^8^. Acute evaluation of hNPs, at four and 24 hours after injury, and the effect on BBB can help determine the extent of direct interaction in the brain. Also, additional studies are needed to determine the therapeutic window after injury.

In conclusion, concomitant lung injury and systemically-mediated vascular injury observed in the brain implies that bTBI has a unique pathology that requires different therapeutics compared to impact-related TBI^23^. Hemostatic nanoparticles are proven effective at increasing survival in a rodent model of blast trauma. In addition to immediate effects, hDNPs improve neurological recovery in the amygdala and mitigate injury pathology. While this study highlights the advantages of hNPs in treating blast injuries, this novel formulation could prove useful in treating a very broad spectrum of traumatic injuries^18,34^. Though more studies are needed to assess the benefits of hemostatic nanoparticles after injury, the results are promising in route to identifying a life-saving and quality of life enhancing treatment for traumatic injury.

## Methods

### Study Design

The purpose of this pre-clinical study was to examine the role of hemostatic nanoparticles loaded with dexamethasone in a rodent blast polytrauma model^47^. Utilizing a side-thorax orientation in the Advanced Blast Simulator (ABS), the rodent sustained a whole body blast exposure^37^. hDNPs were investigated based on their immediate ability to assuage internal hemorrhaging in the lungs, as well as secondary effects on mitigation of neuropathology. The outcomes measured were survival percentages, behavioral assays, and immunohistology. Rodents were randomly placed into treatment groups with the experimenters blinded until statistical analysis was completed. Sample sizes were based on power analyses and previous studies that observed effects using the hemostatic nanoparticles^18,34^. Due to slight variability in experimental blast procedures and the sensitive threshold by which lung injury can cause lethality, a threshold of 26.5 psi static overpressure was applied to exclude animals from the study. This threshold was determined by the 50% lethality range of a weight-adjusted lethality curve established for this polytrauma model^37^.

### Nanoparticle Synthesis and Formulation

Detailed description of nanoparticle synthesis and formulation are described in Hubbard *et al*.^19^. In brief, the copolymer poly(lactic-co-glycolic acid)-poly(ε-cbz-L-lysine)-poly(ethylene glycol) (PLGA-PLL-PEG) was reacted with the peptide GRGDS (control nanoparticles were reacted with a scrambled peptide) in dimethyl sulfoxide (DMSO) and purified by dialysis. The steroid, dexamethasone, was dissolved in acetonitrile at a concentration of 4 mg/mL. Particles for biodistribution were instead made by dissolving the fluorescent marker coumarin (C-6) at 0.2 mg/mL. The block copolymer, PLGA-PLL-PEG-GRGDS, was then dissolved at a concentration of 20 mg/mL in the dexamethasone (or C-6) acetonitrile solution. This solution was added dropwise to a volume of stirring phosphate buffered saline (PBS). Nanoparticles were then collected by coacervate precipitation. The flocculated nanoparticles were then collected by centrifugation. After rinsing, they were suspended in approximately 10 mL deionized water, snap-frozen, and lyophilized for three days. Nanoparticles were resuspended at 20 mg/mL in Lactated Ringer’s (LR) solution and briefly sonicated.

### Nanoparticle Characterization

This section was adapted from Hubbard *et al*.^19^. The diameter of the nanoparticles were characterized using dynamic light scattering (DLS) (90Plus; Brookhaven Instruments) and a scanning electron microscopy (Hitachi S4500). 90Plus software was used to calculate the effective diameter utilizing the DLS data. The PEG corona of the nanoparticles as well as presence of dexamethasone was characterized by NMR (600-MHz Varian Inova NMR spectrometer). Suspension of particles in deuterated water (D_2_O) and again in deuterated chloroform (CDCl_3_) was used to collect data.

### Dexamethasone Release Study

This section was adapted from Hubbard *et al*.^19^. Elution of dexamethasone was garnered by evaluating the release profile of dexamethasone from the particles. Particle samples were incubated at 37 °C in PBS. Supernatant was taken from samples after centrifugation at pre-determined intervals and analyzed using Uv-Vis spectroscopy at 241 nm to determine dexamethasone concentration at each time point. Between time points, the particles were then suspended in additional PBS and returned to the incubator. Supernatant samples were taken over a 13 week time period to achieve long-term release profile of dexamethasone. To determine the total dexamethasone loading of the particles, they were analyzed using UV-Vis spectroscopy. A calibration curve was made by analyzing samples of dexamethasone dissolved in dimethyl sulfoxide (DMSO) at known concentrations. Particles loaded with dexamethasone and control (blank) particles were dissolved in DMSO and compared at 260 nm.

### Experimental Groups and Animal Procedures

Detailed information for experimental procedures can be found in Hubbard *et al*.^19^. The Virginia Tech Institutional Animal Care and Use Committee approved the experimental protocols described herein. In addition, all experiments were performed in accordance with relevant guidelines and regulations. Prior to all experiments, male Sprague Dawley rats (~325 g, Harlan Labs, Frederick, MD) were acclimated to a 12-hour light/dark cycle with food and water provided ad lib. Animals were exposed to a single incident pressure profile resembling a ‘free-field’ blast exposure. The average peak static overpressure of the blast profile was at 27.7 ± 2.29 (BOP ± standard deviation) with positive duration at 2.475 ± 0.16 ms and positive impulse at 21.86 ± 2.00 psi.ms. After animals were excluded based on 26.5 psi threshold, peak static overpressure was 28.49 ± 1.5 psi. All animals were randomly assigned to one of seven groups: Hemostatic Nanoparticles (hNPs), Hemostatic Dexamethasone-loaded Nanoparticles (hDNPs), Control Nanoparticles (cNPs), Control Dexamethasone-loaded Nanoparticles (cDNPs), Lactated Ringer’s (LR), Injury Only (IO) and sham (n = 14–18/group). Prior to blast exposure at 28.5 psi, rats were anesthetized with a ketamine/xylazine solution, in accordance with the rodent weight, for sedation during blast. The shock front and blast overpressure were generated by a custom-built ABS with an end-wave eliminator (ORA Inc. Fredericksburg, VA) located at the Center for Injury Biomechanics at Virginia Tech. The ABS consists of a driving compression chamber attached to rectangular test section chamber with an end-wave eliminator.

A peak static overpressure (28.49 ± 1.5 psi) was produced with compressed helium and calibrated acetate sheets (Grafix Plastics, Cleveland, OH). Pressure measurements were collected at 250 kHz using a Dash 8HF data acquisition system (Astro-Med, Inc, West Warwick, RI) and peak overpressures were calculated by determining wave speed (m/s) at the specimen position. A mesh sling was used to hold the animal during the exposure that allowed for minimal hindrance of the wave through the tube. In addition to holding the animal in a prone position with the right side of the thorax facing the shock wave driver, the animal was prevented from impacting any solid surface to avoid secondary injuries. This was confirmed using high-speed video (Phantom Miro eX2, Vision Research). After blast exposure at 28.5 psi, animals were immediately injected with test solution (hNPs, hDNPs, cNPs, cDNPs, or LR; 5 mg/kg) via tail vein injection. Sham animals underwent all procedures except for blast exposure and tail vein injection.

Survival of animals in all treatment groups was documented and the data was collected for statistical analysis (described below). Animals that survived for three hours after injury typically survived the entire length of the study.

### Behavioral Assays

At two and six days after blast exposure at 28.5 psi, animals underwent an open field thigmotaxis assessment. Briefly, an opaque black acrylic box with dimensions 80 × 80 × 36 cm was used for the task. Animals were acclimated in the open field arena before the injury. The acclimation ensures that anxiety-like traits would be due to the blast and subsequent injury progression. Activity changes were detected using EthoVision XT™ software tracking. Thigmotaxia, preference of proximity to walls, can expose fear of open, lit spaces and is displayed in animals with anxiety. Time spent along the chamber wall reflects an increased level of anxiety. Rats were videotaped for five minutes and avoidance of center square activity (i.e. anxiety-related behavior) was measured by determining the amount of time and frequency of entries into the central portion of the open field. Behavior outliers were excluded based on statistical analysis (±two standard deviations).

### Tissue Processing

At seven days, animals were euthanized by transcardial perfusion of saline and 4% paraformaldehyde. Following collection, brains were stored in a 4% paraformaldehyde fixative solution. After 48 hours in fixative, the whole brains were placed in 30% sucrose solution for tissue sectioning preparation. Whole brains were embedded in Tissue-Tek® optimal cutting temperature (O.C.T.) embedding medium (Sakura Finetek USA, Inc., Torrance, CA) for cryostat processing in the coronal plane. Samples were then cut (40 µm) and sections containing amygdala nuclei were isolated (Bregma: −2.28 mm).

### Lung Histology

Lung processing and H&E staining was performed as previously described^19^. Briefly, lungs were also collected and stored in a 4% paraformaldehyde fixative solution. After 48 hours in fixative, the lungs were placed in 30% sucrose solution for tissue sectioning preparation. Lungs were separated into cassettes with each lobe isolated for analysis and embedded in Tissue-Tek® optimal cutting temperature (O.C.T.) embedding medium (Sakura Finetek USA, Inc., Torrance, CA) for cryostat processing. Samples were then cut (8 µm) and stained with hematoxylin and eosin (H&E) to determine injury extent. Images were taken of three regions of interest (ROI) in each lung tissue section at 10X magnification (Zeiss AxioCam ICc 1). The images were converted to black and white and optical density readings were collected in order to determine the level of hemorrhaging in the lung tissue using ImageJ software (NIH, Bethesda, MD). The percent injured area was calculated in each lobe and significance was determined and reported as mean ± SEM. Lung hemorrhage was quantified by investigators blinded to the treatment groups until statistical analysis.

### Immunohistochemistry

Immunohistochemistry was performed on amygdalar sections (Bregma: −2.28 mm) to evaluate levels of: endothelial barrier antigen (EBA/SMI-71; BBB integrity), glial fibrillary acidic protein (GFAP; activated astrocytes), ionized calcium-binding adaptor molecule 1 (IBA-1; microglia), hypoxia-inducible factor-1α (HIF-1α; hypoxia), vascular endothelial growth factor (VEGF; angiogenesis) and cleaved caspase-3 (C3; apoptosis). Samples were rinsed three times with phosphate-buffered saline (PBS) and incubated in 2% bovine serum albumin (BSA) in PBS for one hour at room temperature. Sections were then incubated with a primary antibody; anti-SMI-71 (EBA) (1:250; Biolegend, San Diego, California), anti-GFAP (1:500; Invitrogen, Carlsbad, California), anti-IBA-1 (1:500; Biocare Medical, Concord, California), anti-HIF-1α (1:250; Novus Biologicals, Littleton, Colorado), anti-VEGF (1:250; Santa Cruz, Dallas, Texas), or anti-caspase-3 (1:500; Cell Signaling Technologies, Danvers, Massachusetts) overnight at 4 °C. After PBS washes, the samples were incubated for 1.5 hours with Alexa Fluor 594 anti-mouse IgG antibody, Alexa Fluor 555 anti-rabbit IgG antibody, Alexa Fluor 488 anti-mouse IgG antibody or FITC anti-rat IgG antibody (Invitrogen, Carlsbad, California). After three PBS washes (five minutes each), samples were mounted, air dried and coverslipped with prolong antifade gold reagent with 6-diamidino-2-phenylindole (DAPI; Invitrogen, Carlsbad, CA). Sections were examined under Zeiss fluorescence microscope at 20X magnification under appropriate fluorescent filters and images were taken by Zeiss AxioCam ICc 1. For all images, quantification (ImageJ software; NIH, Bethesda, MD) was based on fluorescence intensity after thresholding to eliminate background. Immunohistology outliers were excluded based on statistical analysis (±two standard deviations). For immunohistological assays, three brain sections per animal (four images from each section at 20X per sample) were taken and analyzed. Immunohistology for amygdala sections was quantified by investigators blinded to the treatment groups until statistical analysis.

### Fluoro-Jade C Staining

Sections of the amygdala were stained with Fluoro-Jade C (FJC) according to manufacturer’s protocol to identify degenerating neurons regardless of mechanism of cell death (Biosensis, Thebarton, South Australia). Tissue sections were mounted on gelatin coated slides and dried. They were then incubated in a solution of NaOH in 70% ethanol for five minutes. The sections were then transferred to 70% ethanol and distilled water for two minutes each. The sections were then incubated in a solution of potassium permanganate in distilled water and then rinsed in distilled water. They were then incubated in a solution of FJC and DAPI in distilled water. Sections were then rinsed in distilled water thrice, air-dried, and placed on slide warmer until fully dry. The dry slides were cleared in xylene and mounted with DPX (Sigma-Aldrich Co. Ltd, St. Louis, MO). Sections (6 per animal) were examined at 20X on a Zeiss microscope, and analysis was conducted. FJC+ neurons were counted by blinded technicians and the results were quantified.

### Statistical Analysis

Statistical differences between the treatment groups were assessed with analysis of variance, or ANOVA, using LSD post-hoc test where appropriate. All statistical analyses were performed using JMP Pro 10 (SAS Institute, Cary, NC) and p < 0.05 considered statistically significant. Unless indicated otherwise, data are presented as mean ± standard error of the mean, or SEM.

### Data availability

 All relevant data are reported in the article. The datasets generated during and/or analyzed during the current study are available from the corresponding author on reasonable request.
